# Communication successes, challenges, and needs among extension and outreach professionals engaging small-scale and non-intensive swine farmers

**DOI:** 10.3389/fvets.2026.1876400

**Published:** 2026-07-29

**Authors:** Madeline Struck, Michelle L. Schultze, Gabrielle Beckford, Amit K. Pradhananga, Andres M. Perez, Rachel A. Schambow

**Affiliations:** 1 Center for Animal Health and Food Safety, College of Veterinary Medicine, University of Minnesota, Saint Paul, MN, United States; 2 Center for Changing Landscapes, University of Minnesota, Saint Paul, MN, United States

**Keywords:** communication, education, extension, foreign animal disease, non-intensive, pig, small-scale, swine

## Abstract

An introduction of a foreign animal disease (FAD) could greatly harm the United States’ (U.S.) swine industry and cost billions of dollars in losses. Private and public stakeholders have invested heavily in the development of programs to educate swine producers about FAD threats, but their reach to small-scale and non-intensive farmers may be limited. In the U.S., there is a significant contingent of farmers that fall under a wide variety of farm practices, such as small-scale, pasture-raised, and homesteaders. We postulate that research and outreach with these farmers poses unique challenges compared to intensive farmers. To enhance our research-based perspective, agricultural extensionists and educators from 11 U.S. states were interviewed (17 interviewees across 15 interviews) on their experiences working with this audience. Their practice-based perspectives were gathered on the challenges, successes, and ongoing needs of both producers and extension/education professionals. Major challenges included finding this widely dispersed audience, creating engaging materials applicable to diverse production methods and experience, and building relationships with communities that may be distrusting of outside organizations and professionals. Success was found through taking time to listen, adopting communication and education approaches that match specific audience needs, working with trusted partners, and integrating FAD content into broader animal husbandry topics. Ongoing needs include investment of funding and time from industry partners, more personnel, updated educational materials, and a format for broader collaboration. This perspective highlights the needs and opportunities to enhance preparedness and awareness of small-scale and non-intensive swine farming, with the ultimate objective of contributing to mitigate the impact of hypothetical FAD incursions into the U.S.

## 1 Introduction

Small-scale and non-intensive swine farms are a heterogeneous agricultural sector in the United States (U.S.), comprising a wide variety of management styles and production practices that may differ greatly from intensive commercial operations (1). Intensive commercial farms may be generally described as large, integrated swine production systems with increased efficiency of production and usually using specialized stage or age-based production sites and indoor confinement housing (2–4). While there is no uniform definition for “non-intensive,” generally here it is used to refer to farms not using intensive practices for swine production. Large-scale operations with over 1,000 pigs produce the majority of individual pigs, but they account for under 10,000 unique operations nationwide (2). Comparatively, there are over 50,000 operations with 999 or fewer pigs, with 85% of those housing only 1–24 per site. These non-intensive and small-scale operations can be broken out into numerous sub-classifications, including pasture-raised, organic, heritage breed, antibiotic-free, and regenerative, amongst others (1). There is no formal definition for “small-scale” in the U.S., though a previous survey conducted by the U.S. Department of Agriculture in 2021 considered operations with 999 or fewer pigs. The lack of a uniform definition and the audience’s heterogeneity contributes greatly to the challenge of defining the “small-scale” and “niche” audience (5).

With this diversity of practice comes a broad range of motivations for raising pigs, farm priorities and challenges, and farmer communication preferences, especially in terms of biosecurity, disease surveillance, and foreign animal disease (FAD) preparedness. An FAD like African swine fever (ASF) could cause severe animal health and socioeconomic impacts if an introduction occurred. For example, an ASF introduction into the U.S. has projected losses at $50 billion over 10 years (6). Efforts to prevent the incursion of swine FADs into the U.S. have involved extensive collaboration between U.S. public and private industry partners, including preparedness programs such as the Secure Pork Supply Plan (7) and the U.S. Swine Health Improvement Plan (8). However, existing evidence suggests there are significant gaps in FAD awareness, engagement, and, ultimately, capacity for early disease detection amongst small-scale and non-intensive swine farmers. Previous surveys of small-scale U.S. swine farmers reported concerns with veterinary access, a lack of recognition of clinical signs common to FADs, and low levels of participation in both surveillance-oriented diagnostic testing and necropsies following deaths of unknown cause (5, 9–11).

While there are clear gaps in some swine producers’ knowledge of biosecurity and disease management, there are also gaps in our understanding of how to best to conduct research with this group and deliver that education. The U.S.’s Agricultural Cooperative Extension System, composed of outreach and education personnel (extensionists), serves a critical role in providing education on these topics to farmers and ranchers (12). Through years of experience, educators that regularly work with small-scale swine farmers likely hold a wealth of knowledge on the successful strategies and major challenges for reaching and interacting with them. The objective of this work was to use practice-based perspectives to describe and address the need to improve communication and outreach with small-scale and non-intensive swine farmers for research, education, and outreach. By interviewing agricultural extensionists and education professionals already working with this audience, we present a perspective on enhancing research and education activities with small-scale and non-intensive farmers, alongside the strengths and weaknesses of various communication methods around biosecurity and FADs.

## 2 Interviews and data analysis

To inform our perspective, a protocol was developed to conduct interviews with potential experts (described broadly as extensionists, educators, and outreach professionals working with swine farmers) regarding education and communication strategies with target small-scale and non-intensive pig farmers in the U.S. This protocol was approved by the University of Minnesota Institutional Review Board (STUDY00026762). Interview participants were recruited using snowball sampling from December 2025–February 2026. With this method, an initial group of experts are asked to participate and nominate other potential participants; thus, final participant selection is conducted by existing networks and not by the researchers themselves. This reduces researcher-based bias, but may introduce bias toward the existing networks of the interviewees (13). The Swine Education and Outreach Professionals Advisory Committee of the National Pork Board was contacted to initiate the snowball sampling (14). This committee consists of approximately nine professionals that are voted into committee service by swine education and extension professionals. In total, 38 individuals were contacted. The main reason for non-participation was receiving no response (*n* = 13); others gave suggestions for other participants but themselves did not feel they had enough extension or education experience with the target audience. Participants were considered eligible if they were actively working in extension, education, or outreach initiatives with small-scale or related pig farmers in the U.S. Seventeen participants were interviewed across 15 interviews. A consent letter was sent to participants prior to participation.

All interviews were conducted virtually with one to two participants and with one lead interviewer and at least one assistant interviewer. Interviews were conducted from January–February 2026 and took approximately 45 min each. At the start of each interview, the participants gave verbal consent to participate. Interviews were conducted using a semi-structured format, where a predetermined list of questions was used to guide the interview (Supplementary File 1), and follow-up clarifying questions were asked. All interviews were recorded with the participants’ permission.

Transcripts were generated using Zoom’s auto-transcribe feature, cleaned by listening to the recordings, and then coded using NVivo 15.3.1. Interviews were coded by two individuals from the interview team (15). Both deductive and inductive coding was used. For deductive coding, the list of predetermined codes included: challenges, successes, suggestions, needs of interviewee, audience, program topics, and communication strategies. New codes were created in an inductive manner where needed. The coded transcripts were reviewed by the interview team and thematically analyzed to identify major themes (15). Any coding disagreements were resolved through conversation between coders with some input from a third research team member when needed. Member checking was performed by compiling the preliminary thematic analysis and sharing it individually with each participant for their review (16). The final thematic analysis of interviewees’ viewpoints was compiled using participants’ feedback.

## 3 Interview themes

### 3.1 Participant characteristics

Seventeen participants were interviewed across 15 interviews. Participants were from 11 U.S. states (California = 1, Hawaii = 2, Kansas = 1, Maine = 1, Michigan = 1, Minnesota = 2, Missouri = 2, New York = 2, Ohio = 1, Texas = 3, Washington = 1) and from 13 different organizations including universities (*n* = 15), a government agency (*n* = 1), and a state pork association (*n* = 1). Their professional years of experience ranged from 1 to 31 years. The majority had primarily an extension, education, or outreach role (*n* = 15); others (*n* = 2) were primarily a research role but had worked extensively with small-scale swine farmers. The themes identified below represent both the authors’ perspectives and are supported by the interview findings.

### 3.2 Educators’ audience description

Small-scale, niche, and other non-intensive swine farmers are a heterogenous group with different motivations, farming styles, and levels of engagement. They include homesteaders and off-grid farmers seeking self-sufficiency, heritage breeders, 4-H and FFA youth, and show pig farmers. Many raise other livestock species and do not specialize exclusively in swine. This description was strongly supported by the participants’ responses, for example:

“I get the sense that pigs aren't always the main enterprise on the farm, either, for the types of farms that I'm working with” [Anonymous].

Collectively, these farmers vary widely in experience from seasoned producers to new farmers with limited understanding of raising swine. Additionally, farming might not be their primary job or source of income. Many have other jobs, operate seasonally, or raise pigs as a supplemental income source or for personal consumption. This often leads to time constraints, competing priorities, and challenges in extension and education program participation. These farmers may rely on informal or other networks rather than formal swine industry organizations, and some may have limited access to digital resources altogether or prefer not to use this format.

### 3.3 Communication successes

**Figure 1:**
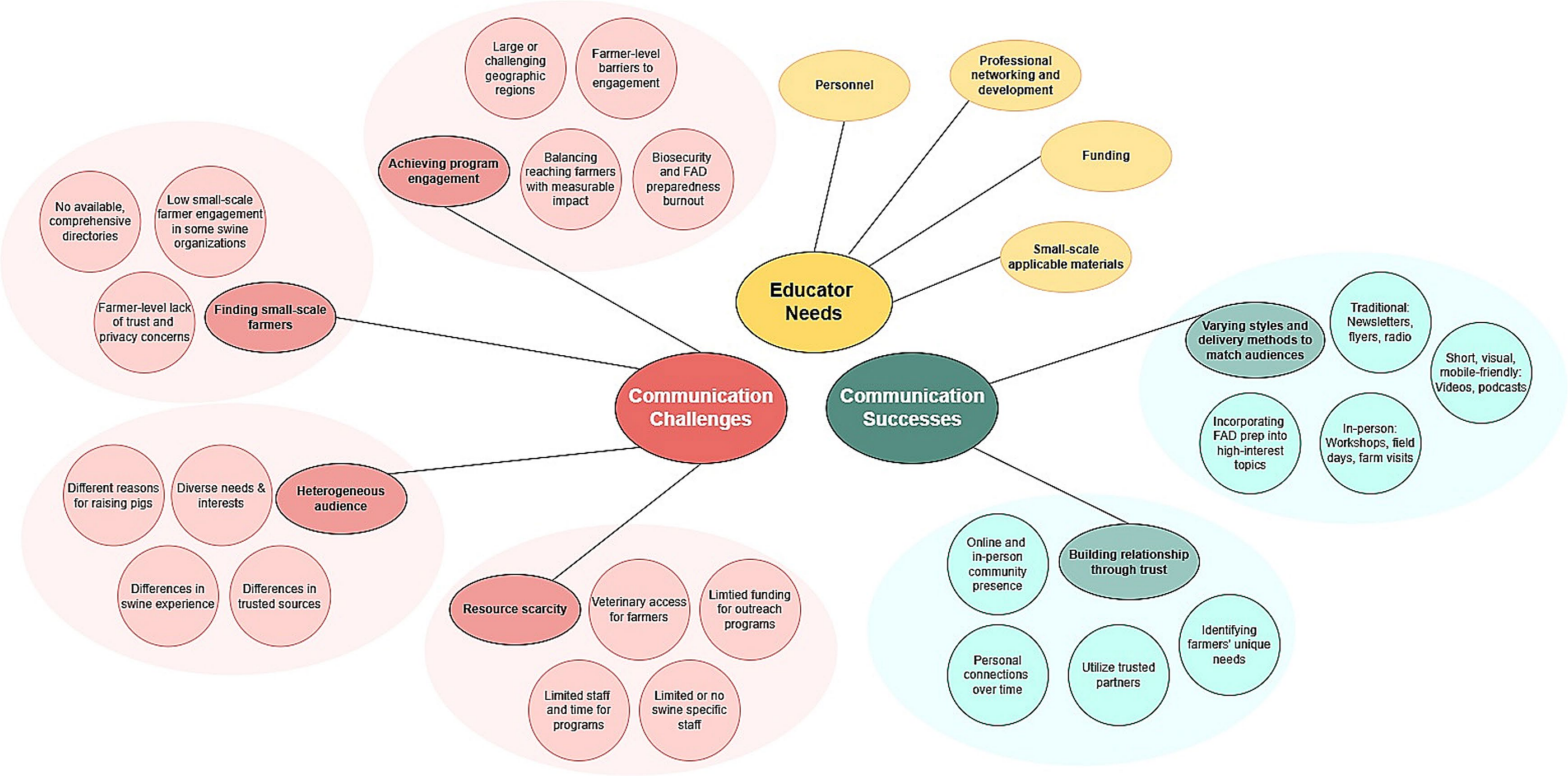
Main themes of communication challenges (red), communication successes (blue), and extension and outreach educators’ needs (yellow) relevant to small-scale and non-intensive swine farmers, as identified through interviews with 17 extensionists and outreach professionals.

Reaching this diverse audience with swine education, particularly on biosecurity and disease preparedness, has long been challenging for governmental personnel, outreach specialists, and researchers. However, the participants identified many successful communication strategies that could inform future work. These included building trust through perseverance and partnerships, using varying communication styles and delivery methods to reach different demographics, creating high-quality educational materials and opportunities for engagement, and strategically presenting topics that may be low-interest to these farmers, like biosecurity or FAD preparedness, in a way that will increase engagement (Figure 1).

Relationship and trust building is vital for reaching small-scale and non-intensive farmers, rather than relying on educational or technical expertise only. For example:

“Usually, if you're the one doing the reaching out to them, you don't get very far. You want to make yourself available, and then when they ask you to… come do a farm visit, or they ask you questions, the most important thing you can do is listen. And do not give any unsolicited advice” [Anonymous].

They emphasized that extension and education is a relationship business, where personal connections through farm visits, phone calls, and local events such as farmers’ and livestock markets are important for engagement. Some participants reported being able to find farmers through social media groups and by attending community-specific events. A key success to learning what farmers need and want has been through taking the time to listen to them, ask questions about their operations, and explore what topics they are interested in. By giving farmers an active role in their own education, educators found more success in creating and delivering programs. Topics that garnered the most farmer engagement were focused on practical swine management and health. Integrating biosecurity and other FAD preparedness into general educational materials, or pairing it with high-interest topics, has been a successful method for avoiding burnout on what can be perceived as an overwhelming subject.

Outreach must be varied to match the diverse target pig farmer population. Common in-person formats used by the participants here included workshops, field days, conferences, and one-on-one farm visits. Many stated that short, visual, and mobile-friendly content was increasingly effective, while also commenting on the continued importance of paper materials, radio, and direct communication for certain audiences. While social media, YouTube videos, online courses, and podcasts can expand reach and be accessible to a wide audience, traditional methods, such as word-of-mouth, feed store flyers, and in-person events, remain important, especially for those less digitally engaged. Interactive programs, including hands-on demonstrations, youth activities, take-home materials (e.g., biosecurity kits), “Ask the Vet” sessions, hands-on veterinary involvement, and incentives such as health screenings have been especially effective for engagement. While not universal, educators reported that many producers trust private and academic veterinarians.

### 3.4 Communication challenges

The major challenges faced in communicating include the heterogeneity of the target audiences, which has been previously described, finding the audience, resource scarcity, achieving program engagement, and establishing trust. Because there is no universal directory of farmers, it is very challenging and time-consuming to find small-scale swine farmers for outreach and research activities, and is further limited by sensitivity and privacy concerns about sharing existing lists. State pork boards and other industry partners may provide some starting points, but not all small-scale swine farmers see value in joining these organizations. From the authors’ perspective and previous experiences, creating sampling frames or lists of small-scale producers requires intensive internet and media searches, which is time-consuming and difficult to replicate or repeat. There is also a subset of farmers that does not want to be found or engaged with, especially by University or government personnel, or who might not consider themselves relevant due to only having swine seasonally. For example:

“And they don't farm year-round, the majority of them. They're seasonal, so it's… If you're looking for, like, a barn. That has pigs year-round, that kind of scale, you're gonna miss a whole bunch” [Anonymous].

Educators, particularly those in extension systems, reported being strained with declines in staff numbers, time availability, and funding constraints. Some felt that industry investment in small-scale farmers was not sufficient to meet ongoing education needs. Some regions, such as the Pacific Northwest or Hawaii, have challenging geography that may force educators to cross mountains or oceans to reach audience members, increasing cost and time needed for in-person program delivery. Additionally, resource allocation for swine programs and swine extension specialists may be underprioritized in some states in favor of more economically-relevant livestock species, leaving large gaps in coverage for swine farmers. Farmers also may have barriers to engagement due to limited time and resources, especially when raising swine is not their primary income source. Many small-scale farmers also lack access to veterinarians and may consult dubious resources for veterinary medical information. Existing educational resources may be underutilized due to lack of effective outreach and producer awareness.

Even when a relationship with the audience is established, engagement with biosecurity and FAD preparedness remains challenging for researchers, animal health officials, and extensionists. The participants reported substantial burnout among small-scale swine farmers, with many suggesting to avoid the word “biosecurity” entirely where possible. Farmers may feel they are too small or isolated to worry about swine disease, or that they do not have an impact on the broader swine industry. Educators reported that in some cases, after an endemic disease outbreak, farmers would abandon swine farming altogether rather than investing their limited resources into improving disease management.

As a result of these challenges, many educators reported that they often feel the effort they put into contacting this audience does not return an equivalent amount of success, making continued attempts hard to justify with their constrained time and personnel, particularly when funding sources or programs require measurable impacts. A similar issue may exist in the academic sphere when considering research with this population. To secure future funding, educators and researchers face a trade-off between reaching many animals through large producers or investing a large amount of time in small-scale farmers who may benefit more individually but represent fewer overall numbers.

### 3.5 Educators’ needs

Participants stated the need for increased funding, staffing, veterinary connections, training, and coordinated information to help improve their outreach to small-scale swine farmers. Funding constraints restrict the ability to host training events, travel to farms, and provide educational opportunities. Staff shortages also leave educators stretched thin and unable to balance creating effective programs, producer outreach, farm visits, and continual communication. This is particularly difficult for states with relatively lower swine production, where extension systems may no longer maintain dedicated swine extensionist positions or strong swine organizations may not be present. A critical gap also exists in veterinary access for small-scale farmers, particularly for veterinarians with swine expertise. Participants expressed a strong need for a directory of veterinarians that work with swine farmers and the creation of materials and training to help non-swine veterinarians feel more comfortable with common swine issues. They also expressed desire for more professional development opportunities, such as marketing techniques to improve their outreach delivery. Additionally, they reported the need for stronger coordination and communication across the industry. While a lot of good information already exists for swine farmers, it is often outdated, not tailored to small-scale farmers, or is difficult to access. Participants highlighted a need for a centralized resource hub that educators can use to provide farmers with reliable, up-to-date materials and a collaborative forum where outreach professionals can share knowledge and strategies for working with these audiences.

## 4 Discussion

This work captures the research-based and education-based perspective of the authors and of extension, education, and outreach professionals, respectively, regarding challenges, needs, and opportunities for communication with small-scale and non-intensive swine farmers. Strategies identified here can enhance awareness and preparedness of this group for disease outbreaks. Major themes from the interviews were the heterogeneity of the target audience and the importance of trust and relationship building (Figure 1). The variety of farmers requires developing a diversity of training materials and delivery methods that motivates these various groups at their different experience levels. Non-intensive swine farmers range from multispecies backyard and seasonal hobby farmers to niche pasture-raised operations and show pig participants, each operating under very different goals and constraints. This creates challenges for educators and research alike; extension efforts must constantly balance between being too general to be useful or too specific to apply broadly, while researchers much balance challenges like the definitions of target populations and interpreting and generalizing results from studies across a heterogenous audience.

For education and outreach, locating and building trust with farmers is a consistent barrier that underpins many of the identified challenges, and success depends on meeting farmers where they are both in the methods of communication and knowledge level. According to the participants, many small-scale farmers are part of closed social or community groups, rely on informal information channels, or intentionally remain hard to reach. A survey of small-scale farmers in the Western U.S. found that they were more likely to source animal health information from the internet (81.8%) than a veterinarian (61.5%) or extension agent (33.9%) (11). Even when they are identified, distrust toward governmental agencies or external experts can prevent their participation. The participants indicated that overall, farmers are more receptive when information comes from individuals they already trust, particularly those who have a personal connection to small-scale or show pig farming. Effective outreach is improved with building relationships and using trusted sources within these communities, but this is time intensive and difficult to continue due to the reduction in funding, personnel, and time.

When considering these findings in the context of FAD awareness and preparedness, many challenges emerge. A one size fits all FAD outreach program that is not delivered by a trusted source will likely have limited success due to the variability in farm types, reasoning for raising animals, and knowledge levels. Characterizing biosecurity strategies that reduce risks in many contexts is also challenging for researchers when farms may vary vastly from site to site. Interest and engagement in FAD-related topics is also likely low; topics such as biosecurity may be more engaging by incorporating them into general husbandry programming, such as disease prevention. For many farmers, swine may represent only a small percentage of household income. Even if they can be convinced of the relevancy of biosecurity to their farm, they may not be able to justify the expense. This is consistent with other studies investigating biosecurity investment across scales of swine farms, where smaller farms were less likely to be classified as biosecurity compliant (17). Outreach programming must take these and other factors into account when trying to promote FAD preparedness amongst these audiences. For future efforts, partnering with veterinarians, state agencies, pork councils, and farmer organizations with established networks can expand reach and credibility, while also helping connect farmers to ongoing support systems. Equally important is how information is delivered. Participants consistently described how producers value practical, experience-based, and hands-on learning over highly technical presentations. Focusing on real-world applications and providing interactive demonstrations can improve retention, trust, and producer participation.

Extensionists and outreach professionals themselves reported many needs. Creating shared repositories of small-scale applicable swine health and FAD preparedness resources, or building awareness of existing resources, will help professionals to efficiently share information with small-scale farmers. Daily work may lead them to feel siloed; dedicated meetings or forums can help build their professional networks and make connections with other professionals who are facing similar challenges. In particular, connecting general livestock extensionists with swine specialists can also help address coverage gaps in states that lack a swine specialist. Improving veterinary availability in rural areas would also help lift the education burden. Extensionists frequently reported being asked to fill the gaps left by lack of veterinary access, with 23.2% of U.S. counties designated as lacking food animal veterinarians (18). While extensionists face ongoing budget cuts and shortages, building the capacity and strength of existing personnel and their networks can help to address ongoing challenges with swine health and FAD preparedness.

There are multiple limitations to the work presented here. As with any perspectives work, this represents the perspectives of the authors’, though is supported by the practice-based perspectives of extensionists and educators. However, this is a small sample size of extensionists and educators (17), and we cannot rule out that others outside of our consideration would have differing opinions. The nature of snowball sampling could have biased our analysis toward like-minded individuals as well. Additionally, the farmers themselves were not interviewed, and their perception of the effectiveness of their communications and own needs may differ from the participants.

As the need for swine health and FAD preparedness persists, researchers, extensionists, and other professionals that work directly with farmers will continue to become increasingly important. Here, the perspectives of extensionists and educators were captured, highlighting the need for continued support from public and private partners to deliver critical animal health information to farmers. While based on the subjective opinions of these individuals, the themes identified from the interviews were consistent across U.S. regions, niches, and experience levels of the audience being served. This work was specific to swine farmers; however, these findings are likely applicable to many types of small-scale farmers. Future work should continue to explore innovative approaches for connecting with small-scale farmers and developing materials that are applicable and appropriate to their needs.

## Data Availability

The raw data supporting the conclusions of this article will be made available by the authors, without undue reservation.
