# Supplementary material for: Communication successes, challenges, and needs among extension and outreach professionals engaging small-scale and non-intensive swine farmers
Source: Front Vet Sci. 2026 Jul 29;13:1876400. doi: 10.3389/fvets.2026.1876400 (PMC13461530; doi:10.3389/fvets.2026.1876400)
Supplement: Supplementary file 1 [file Table_1.DOCX]

**Supplementary File 1. Predetermined list of questions used to guide interviews with swine extension and education specialists.**

1. What is your current professional role?
2. How many years have you worked in your field?
3. What state(s) do you currently work in?
4. Please tell us a little about your current role and the types of education/extension work that you do, such as the types of farmers you commonly work with or the types of topics you teach.
   a. What are some of your goals related to your outreach or research work?
5. What have been helpful strategies or successes in communicating or conducting education to small-scale, niche, beginning, organic, and other types of non-intensive swine farmers and owners in your area?
   a. What makes/made that strategy successful?
   b. How did you evaluate/determine that it was successful/what do you consider success?
6. What are the challenges that you have in communicating or conducting education with these farmers?
   a. How are these different or similar from other groups you work with?
   b. Have you found strategies to address that challenge?
7. Have you conducted any type of training or outreach with these types of farmers regarding foreign animal disease preparedness, such as biosecurity or how to recognize disease?
   a. Have you experienced or do you perceive any particular challenges with educating on FAD content to this audience?
   b. Would you be interested in developing more/do you see a need for it with this audience?
   c. What types of topics do you think are most needed? / If you had to recommend someone to start with any FAD prep topic, what would start with it?
   d. If you haven’t done any FAD prep education, why not?
8. What resources, tools, or training would be most helpful to you in increasing your ability to reach these types of farmers and to address the challenges you mentioned? / What do you need to do your job better?
9. Anything else you would like to share about your work/lessons learned?
10. Is there anyone else you think we should talk to about this topic?
